# Cytotoxicity of Two Experimental Epoxy Resin-Based Sealers 

**DOI:** 10.22037/iej.v13i2.19530

**Published:** 2018

**Authors:** Hengameh Ashraf, Farhood Najafi, Soolmaz Heidari, Zahra Yadegary, Saeede Zadsirjan

**Affiliations:** a *Department of Endodontics, Dental School, Shahid Beheshti University of Medical Sciences, Tehran, Iran; *; b *Department of Resin and Adhesives, Institute for Color Science and Technology, Tehran, Iran; *; c *Department of Dental Biomaterials, Dental School, Tehran University of Medical Sciences, Tehran, Iran*

**Keywords:** Cytotoxicity, Endodontics, Epoxy Resin, Sealer

## Abstract

**Introduction:**

Many endodontic sealers are available, but search for the ideal sealer continues. This study aimed to assess the cytotoxicity of two experimental endodontic sealers in comparison with AH-26 resin sealer.

**Methods and Materials:**

This *in vitro* study was conducted on conventional and experimental root canal sealers: AH-26, an epoxy resin experimental sealer A (ES-A) composed of calcium tungstate, zirconium oxide, aerosil, bismuth oxide, titanium oxide, hexamine and an epoxy resin and experimental sealer B (ES-B) with compositions similar to ES-A except for the presence of imidazoline as a catalyst. The experimental sealers containing nano-particles were mixed with 37.5% of an epoxy resin. The extraction of five samples of each experimental sealer (A, B) and AH-26 sealer were subjected to MTT assay in the form of set and fresh at 1, 24 and 72 h with 1, 10, 100% dilution according to the International Standard ISO:10993-2012. Data were analyzed using the one-way ANOVA.

**Results:**

The set ES-A had the least cytotoxicity from the first hour but the cytotoxicity of ES-B and AH-26 extraction decreased over time. In fresh form, except for 100% concentration, ES-A showed the least cytotoxicity compared to the other two sealers.

**Conclusion:**

All three sealers had high cytotoxicity in 100% concentration but had low cytotoxicity in 10% and 1% concentrations.

## Introduction

Biomechanical cleaning of the root canal system is one major goals of endodontic treatment. Proper instrumentation, irrigation and intracanal medicaments can significantly decrease the count of microorganisms in an infected root canal. However, The presence of residual bacteria in dentinal tubules has been demonstrated [1, 2]. 

Cleaning and shaping of the root canal system is performed to prepare the apical region for root canal filling. Root filling after cleaning and shaping is an important step of endodontic treatment. Long-term success of endodontic treatment can be achieved by proper three-dimensional filling of the root canal system and appropriate coronal restoration to provide a seal and prevent bacterial leakage [3, 4]. During the filling stage of root canal treatment, periradicular tissues may contact endodontic sealers mainly by extrusion through the apical foramen [5]. When sealers are in intimate contact with the periapical tissues for extended periods of time, their breakdown toxic products may hamper the periapical healing process by inhibiting the proliferative capability of the periradicular cell population [6, 7]. Therefore, apart from good physical and chemical characteristics, endodontic sealers should be biologically compatible [8-10].

Several methods have been recommended for filling of root canals. Use of gutta-percha, a semi-solid root filling material, in combination with sealer is the most commonly performed method of root filling. Gutta-percha alone is not suitable for root filling because it does not have adequate flow and does not adhere to the root canal wall [11]. 

**Figure 1:**
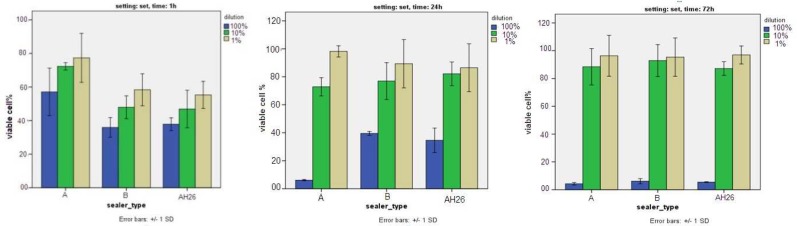
Percentage of viable cells in the experimental groups in presence of different concentrations of set sealers at 1, 24 and 72 h

**Figure 2:**
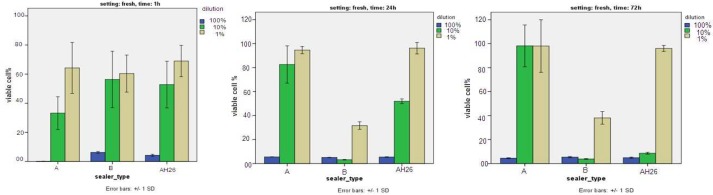
Percentage of viable cells in the experimental groups in presence of different concentrations of fresh sealers at 1, 24 and 72 h

A hermetic seal cannot be achieved without using a sealer. An ideal sealer must flow along the root canal wall and fill the gaps between the gutta-percha and canal wall to decrease the risk of endodontic treatment failure [12].

A previous study assessed physical properties and characteristics of some experimental endodontic sealers according to the International Standard ISO for dental root canal sealing materials 6876 [2]. This study aimed to assess the cytotoxicity of experimental endodontic sealers in comparison with AH-26.

## Materials and Methods

The study was conducted on conventional and experimental root canal sealers: AH-26 (Dentsply, De Trey, Konstanz, Germany), an epoxy resin experimental sealer (ES-A) composed of calcium tungstate, zirconium oxide, aerosil, bismuth oxide, titanium oxide, hexamine and an epoxy resin (Sigma-Aldrich, St Louis, MO, USA) and ES-B with compositions similar to ES-A except for the presence of imidazoline as a catalyst [2]. The experimental sealers containing nano-particles were mixed with 37.5% of an epoxy resin. The powder/liquid ratio of ES-A and ES-B sealers were determined by a pilot study that tested several times with different percent of composition of powder and liquid. Then, we selected two powders and one liquid. Finally, after testing the physical, chemical, mechanical and cytotoxicity of experimental sealers, we choose the best powder. AH-26 (Dentsply, De Trey, Konstanz, Germany) was mixed according to manufacturer’s instructions. 

***Cell culture***

Cell culture flask of L929 murine fibroblast cell line was obtained from the National Cell Bank (Pasteur Institute of Iran). After culture, the 4^th^ passage cells were detached using trypsin-EDTA (Gibco, USA).

After ensuring cell viability (over than 95%) by using the standard Trypan Blue uptake technique [13], the cells were counted using a hemocytometer (Neubauer Improved Bright-Line Chamber, Precicolor HBG, Germany). A total of 5000 cells were seeded in each well of a 96-well plate as mono-layer. Three plates were used for each material to be tested (for assessment at one, 24 and 72 h). 

The cells were cultured in complete media included Dulbecco’s modified Eagle’s medium (DMEM) (Life Technologies, Inc., Grand Island, NY, USA) supplemented with 10% fetal bovine serum (Gibco, USA), 100 IU/mL penicillin (Sigma-Aldrich, St. Louis, MO, USA) and 100 µg/mL streptomycin (Sigma-Aldrich, St. Louis, MO, USA). The cell culture plates were incubated at 37° C, 98% humidity and 5% CO_2_ for 24 h.

***Preparation of materials***

The three sealers were prepared under sterile condition in laminar flow hood and applied to the wells of a 6-well plate (Cell star, Greiner bio-one). After mixing, they were coated at the bottom of the wells with a minimum of 1 mm thickness. Sealers were then incubated at 37°C and 98% humidity for 48 h. After 48 h, the three sealers were mixed again and coated to the bottom of the wells to serve as the fresh groups.

***Preparation of extract***

According to ISO 10993-12:2012 standard, culture medium (DMEM) was added to the coated 6-well plates. The plates were incubated at 37°C for 24 h. After 24 h, the extracts were sterilized by filtering (0.22 µm pore size, Schleicher & Schuell; Germany). Then the extracts were supplemented with 10% FBS and 100 IU/mL penicillin and 100 µg/mL streptomycin. To observe dose-dependent responses, samples were diluted with completed media (1%, 10% and 100% concentrations).

***Exposure of cells to the extracts***

The extracts were adjusted for pH in 7.2-7.4 before exposure. Then, three 96-well plates containing cells were used to expose the cells to each extract. Each plate was allocated for one assessment time point (1, 24 and 72 h). The test for concentrations of each group was repeated five times. Culture medium of each well was removed and then 200 µL of prepared extracts (different concentrations) were replaced to each well. In positive control group, distilled water was used instead of extract or culture medium [14-16], and in negative control group, complete media was used instead of extract [17]. After exposure of cells to the extracts, the plates were incubated at 37°C, 98% humidity and 5% CO2, for 1, 24 and 72 h.

***MTT assay***

After 1, 24 and 72 h incubation time, the supernatants were removed from wells and the sterile MTT solution (5mg/mL) diluted in cell culture media (1:10 ratio) were replaced then incubated at 37°C, 98% humidity for 2 h. After that, the formazan crystals were dissolved in dimethyl sulfoxide. Optical density was then read using ELISA Reader (Anthos 2020.Austria) at 570 nm wavelength with 620 nm reference.

***Data analysis***

The mean and standard deviation of each variable were reported in AH-26 sealer and experimental sealer groups (ES-A and ES-B). One-way ANOVA was used to compare the mean of each variable among three groups. Tukey’s test was used for pairwise comparisons. 

## Results

***Set sealers***

The results of 1 h exposure, in all three concentrations of 1%, 10% and 100%, showed that ES-A had significant higher cell viability (*P*<0.05). 

The results of 24 h exposure, in 100% concentration, showed that ES-A had significantly higher cytotoxicity (*P*<0.001) while in 10% and 1% concentrations, no statistically significant difference was noted among the sealers (*P*>0.05). 

The results of 72 h exposure, in all three concentrations of 1%, 10% and 100%, no significant difference was noted among three sealers (*P*>0.05) and all three showed high cytotoxicity in 100% concentration (Figure 1). 

***Fresh sealers***

Based on the results of 1 h exposure, significant cytotoxicity was noted for all three sealers only in 100% concentration (*P*<0.05). The results of 24 h exposure, showed that all three sealers had significant cytotoxicity in 100% concentration (*P*<0.05). In 10% concentration ES-A, AH-26 and then ES-B showed the highest cell viability, respectively. In 1% concentration, ES-B showed the lowest cell viability compared to ES-A and AH-26 (*P*<0.001). 

After 72 h exposure, comparison of the three sealers in 100% concentration showed significant cytotoxicity (*P*<0.05). At this time point, in 10% concentration, ES-A showed the highest cell viability (*P*<0.001). In 1% concentration, ES-B showed the lowest cell viability compared to ES-A and AH-26 (*P*<0.001) (Figure 2). 

## Discussion

This study assessed the cytotoxicity of A and B experimental endodontic sealers in comparison with AH-26 sealer in 100%, 10% and 1% concentrations after 1, 24 and 72 h of exposure of murine fibroblasts. 

The experimental endodontic sealers evaluated in this study had a resin base with physical and chemical characterization evaluated in previous study [2]. The reason behind their selection was the fact that despite the introduction of new MTA- and silicon-based sealers as well as ceramic sealers with calcium-silicate-phosphate base, resin sealers are still used in dental clinics due to high radiopacity, dimensional stability, low solubility, low linear expansion, high flow ability, good bond to dentin, easy handling and easy retreatment [18]. 

Extrusion of endodontic sealers through the apical foramen is unfavorable due to direct contact of sealer with the periapical tissue. Sealers affect the immune cells and cause inflammation of the periapical area and thus, affect the clinical success of treatment [19]. 

Many parameters characterize the biocompatibility of endodontic sealers, such as genotoxicity, mutagenicity, carcinogenicity, histocompatibility and immunological affects [20]. Several studies have focused on the cytotoxicity of endodontic sealers [9, 21-25], and some on genotoxicity [6, 9, 10, 22, 26-31].

Evaluation of cytotoxicity is the first test for assessment of biocompatibility of dental materials. Normally, extract of materials is tested by dissolution in the culture medium [32]. The MTT assay is commonly used for assessment of cytotoxicity of dental materials. The MTT assay is a colorimetric test for assessment of the number of viable cells. [33]. However, it should be mentioned that this test, similar to other laboratory tests, has limitations such as absence of defense and inflammatory mechanisms and absence of cellular interferences similar to those occurring *in vivo*; this makes generalization of results to the clinical setting difficult [33]. 

In the MTT assay, direct and indirect methods are used to expose cells to materials [17, 20, 34, 35]. In the indirect method, an extraction vehicle is used and the cells are exposed to the released extract. We used the indirect method in this study. Since this study evaluated endodontic sealers, selection of extraction method was appropriate because after endodontic treatment, some compounds leach out from the root canal filling material into the periapical tissue and indirectly affect the cells [17, 36-39]. 

In the pilot study, pure extract of AH-26 decreased cell viability by about 34.6±8.8% in the first 24 h, which was in line with the results of Gerosa *et al. *[40], Huang *et al. *[6] and Javidi *et al. *[41]. In the main study, in the presence of the extract of set sealers in 1% and 10% concentrations, all three sealers showed low cytotoxicity and their cytotoxicity decreased over time. But in 100% concentration, all three sealers showed very high cytotoxicity at 1, 24 and 72 h. In fresh form, except for 100% concentration, the ES-A showed the least cytotoxicity compared to the other two sealers. The cytotoxicity of the ES-A in contrast to ES-B decreased over time when used in 1% and 10% concentrations but cytotoxicity of AH-26 sealer only in 1% concentration decreased over time.

Based on previous studies, direct contact with pure AH-26 extract significantly decreases cell viability [34, 42-46]. Animal studies reported that the destructive effects of these materials on viable cells are limited. Inflammatory reactions along with blood circulation in the process of tissue healing decrease the primary cytotoxicity of materials [32]. Thus, 1%, 10% and 100% concentrations of extracts were tested in our study.

The degree of cytotoxicity changes with the degree of setting and dilution of materials [45]. The significance of biocompatibility of endodontic sealers is highlighted when in direct contact with periapical tissue because the released or degraded materials may have adverse effects on the surrounding tissue. 

The current results showed that all tested sealers had some degrees of cytotoxicity, which were considerably high in fresh form. The fresh application of sealers well simulates the clinical setting since sealers are applied to the canal wall in freshly mixed form and later set in the canal. Thus, they have a relatively high biological risk compared to other dental materials [47]. Within the limitations of this study, the results of cytotoxicity test showed that sealers had high cytotoxicity at first but in set form and in 10% and 1% concentrations, their cytotoxicity decreased over time, which may indicate that a large amount of the extract is released at first but over time, release of cytotoxic compounds decreases into the cell culture medium. 

Cytotoxicity depends on the concentration, time lapse after mixing, the test type, the cell type used or the sealer being fresh or set [10]*.* All three materials in this study showed decreased cytotoxicity after setting. It should be noted that *in vitro* analysis assessed the cytotoxicity of the material after setting, whereas in *in vivo* study, the sealer used were still fresh. It is known that fresh or set sealers can cause different reactions in cell, and/or tissues [39]. Several *in vitro* and *in vivo* studies reported that root canal sealers with epoxy resin base in fresh and set forms had the ability to induce high cytotoxic effects [48-53]. These experimental evidences have been clinically confirmed as well [44].

The formaldehyde released from sealer in the process of setting is responsible for cytotoxicity of AH-26 especially in the first hours after polymerization [17, 45]. AH-26 sealer contains hexamethylenetetramine, which breaks down into ammonia and formaldehyde. The amount of formaldehyde released from AH-26 and AH-Plus is 1347 and 3.9 ppm, respectively [54]. AH-26 liquid contains bisphenol-A-diglycidylether. Schweikl *et al.* [49] attributed high cytotoxicity of AH-26 to epoxy-bisphenol A in its composition. They believed that liquid is an active component of sealer and cytotoxicity of AH-26 is not due to the products of its setting reaction such as formaldehyde [49]. Despite significant cytotoxic effects of AH-26 sealer, it is routinely used in the clinical setting. It should be noted that if a material is toxic *in vitro*, it may not necessarily show high cytotoxicity *in vivo*. Thus, *in vivo* studies are required to assess the biocompatibility of these sealers and their effects on success of endodontic treatment. 

## Conclusion

Original extracts presented the highest cytotoxicity activity in fresh and set forms. The tested sealers did not present expressive cytotoxic levels in more diluted samples. The cytotoxicity of the ES-A in contrast to ES-B decreased over time when used in 1% and 10% concentrations but cytotoxicity of AH-26 sealer only in 1% concentration decreased over time.
